# The Effect of Glycine and N-Acetylcysteine on Oxidative Stress in the Spinal Cord and Skeletal Muscle After Spinal Cord Injury

**DOI:** 10.1007/s10753-023-01929-9

**Published:** 2023-11-17

**Authors:** Xin Xu, Chun-Jia Zhang, Zuliyaer Talifu, Wu-Bo Liu, Ze-Hui Li, Xiao-Xin Wang, Hua-Yong Du, Han Ke, De-Gang Yang, Feng Gao, Liang-Jie Du, Yan Yu, Ying-Li Jing, Jian-Jun Li

**Affiliations:** 1 School of Rehabilitation, Capital Medical University, Beijing, 100068 China; 2 Department of Spinal and Neural Functional Reconstruction, China Rehabilitation Research Center, Beijing, 100068 China; 3 Chinese Institute of Rehabilitation Science, Beijing, 100068 China; 4 Center of Neural Injury and Repair, Beijing Institute for Brain Disorders, Beijing, 100068 China; 5 Beijing Key Laboratory of Neural Injury and Rehabilitation, Beijing, 100068 China; 6 School of Population Medicine and Public Health, Chinese Academy of Medical Sciences/Peking Union Medical College, Beijing, 100730 China; 7 Cheeloo College of Medicine, Shandong University, Jinan, 250100 Shandong Province China; 8 Department of Orthopedics, Qilu Hospital of Shandong University, Jinan, 250100 Shandong Province China; 9 School of Rehabilitation Sciences and Engineering, University of Health and Rehabilitation Sciences, Qingdao, 266000 Shandong Province China

**Keywords:** spinal cord injury, GlyNAC, oxidative stress, skeletal muscle atrophy, glutathione, secondary injury.

## Abstract

Oxidative stress is a frequently occurring pathophysiological feature of spinal cord injury (SCI) and can result in secondary injury to the spinal cord and skeletal muscle atrophy. Studies have reported that glycine and N-acetylcysteine (GlyNAC) have anti-aging and anti-oxidative stress properties; however, to date, no study has assessed the effect of GlyNAC in the treatment of SCI. In the present work, we established a rat model of SCI and then administered GlyNAC to the animals by gavage at a dose of 200 mg/kg for four consecutive weeks. The BBB scores of the rats were significantly elevated from the first to the eighth week after GlyNAC intervention, suggesting that GlyNAC promoted the recovery of motor function; it also promoted the significant recovery of body weight of the rats. Meanwhile, the 4-week heat pain results also suggested that GlyNAC intervention could promote the recovery of sensory function in rats to some extent. Additionally, after 4 weeks, the levels of glutathione and superoxide dismutase in spinal cord tissues were significantly elevated, whereas that of malondialdehyde was significantly decreased in GlyNAC-treated animals. The gastrocnemius wet weight ratio and total antioxidant capacity were also significantly increased. After 8 weeks, the malondialdehyde level had decreased significantly in spinal cord tissue, while reactive oxygen species accumulation in skeletal muscle had decreased. These findings suggested that GlyNAC can protect spinal cord tissue, delay skeletal muscle atrophy, and promote functional recovery in rats after SCI.

## Introduction

Spinal cord injury (SCI) is a serious medical condition that can severely disrupt the normal functioning of the central nervous system. The occurrence of a primary injury is often followed by a series of secondary injuries, including disruption of the blood–spinal cord barrier, neuroinflammation, and oxidative stress (OS)-induced damage, among others, and this phase may last for extended periods [1, 2]. Studies have confirmed that OS is an important cause of injury exacerbation after SCI. OS refers mainly to an imbalance between oxidation products and the antioxidant system. The microenvironment of the injured spinal cord is characterized by mitochondrial metabolic imbalances, leading to the excessive production of free radicals and the consequent peroxidation of cell membrane lipids, which results in spinal cord nerve cell damage and even death [3–5].

Impaired central nervous system regulation following insult to the spinal cord leads to abnormal signaling in peripheral nerves that innervate skeletal muscle, resulting in skeletal muscle atrophy [6, 7]. Skeletal muscle atrophy is a frequently occurring complication of SCI characterized by decreased skeletal muscle mass, strength, and endurance [8]. Skeletal muscle atrophy results from mitochondrial toxicity in denervated skeletal muscle, which can impair the scavenging of reactive oxygen species (ROS), leading to oxidative injury and the apoptosis of skeletal muscle cells [9, 10]. Therefore, alleviating oxidative stress-related injury in the spinal cord and skeletal muscle represents an important strategy for treating skeletal muscle atrophy after SCI.

Glutathione (GSH), the most abundant non-protein thiol in cells, is an endogenous antioxidant with a key role in the regulation of oxidative stress [11]. The levels of this antioxidant are greatly depleted after SCI and cannot be replenished *via* endogenous synthesis [12]. GSH is synthesized from glutamate, glycine, and cysteine. After SCI, glutamate release increases, resulting in cytotoxicity [13], while glycine and cysteine levels are relatively decreased [14, 15]. Accordingly, we hypothesized that an exogenous supply of glycine and cysteine might lead to increased GSH synthesis *in vivo*, as well as the maintenance of the levels of metabolism of these essential amino acids. Glycine and N-acetylcysteine (GlyNAC), a GSH precursor complex, can exogenously replenish glycine and cysteine levels and was reported to attenuate oxidative stress-induced damage and delay aging [16]. However, no study to date has assessed the therapeutic effect of GlyNAC in SCI. Thus, the aim of this study was to investigate the effects of GlyNAC intervention on oxidative injury in the spinal cord and skeletal muscle of rats with SCI and identify the putative underlying mechanisms.

## Materials and Methods

### Animals and Groups

**Fig. 1:**
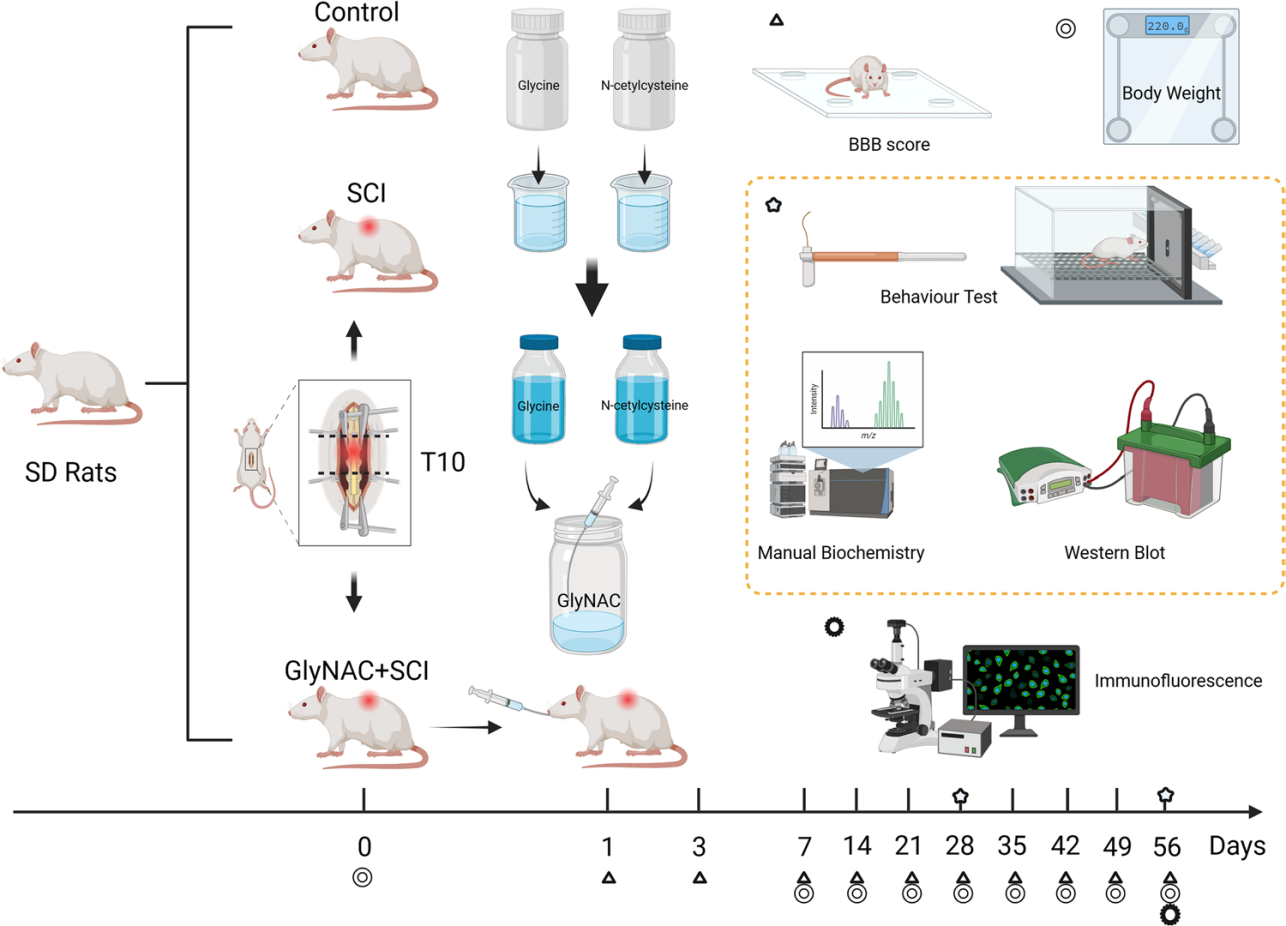
Experimental procedure and time points. The rats were randomly divided into three groups, in which the SCI group and the GlyNAC+SCI group underwent modeling surgery for spinal cord injury at the level of T10. GlyNAC was administered by gavage 1 day after modeling and BBB scores were assessed on days 1 and 3 after modeling to determine if modeling was successful. The body weight of the rats was measured once before modeling and then once a week for 8 consecutive weeks after modeling. Biochemical tests, behavioral assessments, and western blotting assays were performed at 4 and 8 weeks after injury while immunofluorescence staining assays were performed at 8 weeks. BBB, Basso–Beattie–Bresnahan; GlyNAC, glycine and N-acetylcysteine; SCI, spinal cord injury.

In this study, a total of 50 female Sprague–Dawley (SD) rats (female rats were selected as their urethra is short and wide, which facilitates their care after SCI), 10 weeks old, weighing 220 ± 20 g, were purchased from Specific Biotechnology Co., Ltd (Beijing, China; License No.: SCXK[Beijing]2019-0010). The animals were randomly divided into 3 groups—a control group (no treatment, *n* = 10), a SCI group (modeling of severe SCI at the T10 level, *n* = 20), and a GlyNAC+SCI group (modeling of severe SCI at the T10 level plus GlyNAC intervention by gavage, *n* = 20) (Fig. 1).

### GlyNAC Preparation

Glycine (Catalog No. 410225) and N-acetylcysteine (Catalog No. A7250) were purchased from Sigma-Aldrich Trading Co., Ltd. (Shanghai, China). Glycine and N-acetylcysteine were dissolved in the daily drinking water of the rats. The glycine stock solution was stored at room temperature and the N-acetylcysteine stock solution was stored at 4 °C. The two solutions were mixed in a 1:1 ratio 10 min before use.

### SCI Modeling Preparation and Gavage Intervention

The rats were anesthetized with isoflurane and were placed flat in a prone position on an insulating mat. After shaving and sterilizing, the skin and muscles in the back of the animal at the level of T10 were cut longitudinally to fully expose the vertebral plates and pedicles of T9–T11. The T10 vertebral plate was excised to expose the spinal cord of the T10 segment, which was then rinsed and moistened with saline. The T9 and T11 vertebral plates of the rats were subsequently clamped, and the rats were placed on a spinal cord percussion device (Precision Systems and Instrumentation IH Spinal Cord Percussion Device, USA) with the percussion force set to 250 kdynes, resulting in a moderate SCI model. Spasticity in the tail swing of the rats could be observed after the blow, following which the surgical incision was sutured, and 1.5 mL of saline, as well as 1 mL of penicillin solution, was injected subcutaneously for rehydration and antimicrobial treatment. After surgery, the rats were placed in an incubator and were returned to the rearing cage box when they woke up.

GlyNAC intervention (200 mg/kg, pre-experimentally determined) started at 10:00 h on postoperative day 1. After mixing, the GlyNAC solution was administered *via* a gavage needle (Zhongke Hengtian, Beijing China). Body weight was assessed once a week and the dosage was adjusted according to the change in body weight. Gavage was performed for 4 weeks in total. Bladder care was carried out once every 12 h and the rats were helped with urination for approximately 1–2 weeks. If the rats had hematuria, penicillin injection was continued and the animals were closely monitored (Fig. 1).

### Basso–Beattie–Bresnahan (BBB) Locomotor Rating

The BBB score ranged from 0 to 21. Locomotor function in the model rats was assessed using BBB scores on days 1 and 3 after modeling. If the rats showed any movement of the lower limbs (score > 0), they were excluded from the experiment. Two observers blinded to the experimental grouping were selected to evaluate the BBB scores and complete the recordings after modeling. The rats were allowed to move freely in an open field for 15 min and the two observers scored and recorded the scores once a week for 8 consecutive weeks (Fig. 1).

### Body Weight Determination

The body weights of the rats were assessed before modeling (day 0) and for 8 consecutive weeks after modeling. Changes in body weight were calculated by subtracting the preoperative body weight from the body weight assessed in each postoperative week. During the first 4 weeks, the dosage of the intervention was adjusted for each rat according to the change in body weight of each of the postoperative rats.

### Assessment of Mechanical and Thermal Pain

Mechanical pain in rats was assessed using the Von Frey method. The rats were individually placed in a plastic cage with a metal mesh floor and the tests were performed after 15 min of acclimatization. The bottom of the rat’s hind paw was stimulated with Von Frey hairs of different thicknesses and the pain response was scored on the Von Frey Mechanical Pain Scale. If the rats had a violent reaction, such as foot shrinkage, foot lifting, and foot licking, then the rats were considered to have developed pain, which was labeled as “O”; otherwise, the rats were considered to have not developed a pain reaction, which was labeled as “X.” The interval between each stimulus was 5–10 min. For thermal pain, the hot plate method was used (Bioseb, Pinellas Park, FL, USA), in which the rats were placed on a plate heated to 50 °C and the time it took for the rats to lift or lick their feet was recorded. The thermal pain test was stopped if the time exceeded 30 s to prevent the paw from being burned. The average of three recordings was obtained for each (left and right) hind paw, with an interval of 10 min between each recording.

### Determination of Superoxide Dismutase (SOD) Activity

We applied the hydroxylamine method to measure SOD activity. SOD activity was measured in T10 spinal cord samples on days 28 and 56 after injury using a SOD kit (A001-1, Nanjing Jianjian Bioengineering Institute, Nanjing, China) according to the manufacturer’s instructions. Once homogenized samples had been thoroughly mixed with the reagents, the samples were incubated in a water bath at a constant temperature of 37 °C for 40 min. The color developer was then added and the OD value of the supernatant was measured at 550 nm using a microplate reader (Epoch, BioTeK). The protein concentrations of the spinal cord samples were also determined and a standard curve was plotted to calculate relative SOD activity.

### Determination of Malondialdehyde (MDA) Concentrations

We used the TBA method to determine the concentration of MDA. MDA concentrations in T10 spinal cord samples were determined on days 28 and 56 post-injury using an MDA kit (G4300, Servicebio). The tissues were first homogenized and lysed, and, after standing, the supernatant was mixed with the assay solution and incubated at 95 °C for 40 min, transferred to an ice bath for 5 min, and then centrifuged at 10,000 × *g* for 10 min. Finally, the OD of the supernatant at 532 nm was detected using a microplate reader.

### Determination of the Total Antioxidant Capacity (T-AOC)

T-AOC was measured in gastrocnemius muscle samples on days 28 and 56 after injury using a T-AOC kit (A015-2, Nanjing Jianjian Bioengineering Institute) and the ABTS method. The tissue was first homogenized and lysed, and, after standing, the supernatant was added to the reagent and left to stand at room temperature for 6 min. Subsequently, the OD of the supernatant at 405 nm was detected in a spectrophotometer. Simultaneously, the protein concentrations of the gastrocnemius muscle samples were measured to calculate the T-AOC.

### Measurement of the GSH Concentration

For the determination of GSH concentration, we used the microplate test method. GSH concentrations were measured in gastrocnemius muscle and T10 spinal cord samples on days 28 and 56 after injury using a GSH kit (A015-2, Nanjing Jianjian Bioengineering Institute) and the ABTS method. Once the tissues had been homogenized and lysed, the reagents were mixed and centrifuged at 3500 rpm for 10 min. The supernatant and the other reagents were then mixed and left to stand for 5 min. The OD of the supernatant at 405 nm was detected using a microplate reader, and the protein concentrations of the spinal cord and gastrocnemius muscle samples were determined to calculate the GSH concentration using the following formula: $$GSH\ concentration=\left(assay\ OD\ value-blank\ OD\ value\right)$$$$/\left(standard\ OD\ value-blank\ OD\ value\right)$$$$*standard\ tube\ concentration \left(20\ \upmu \mathrm{mol}/\mathrm{L}\right)$$$$*sample\ pretreatment\ dilution \left(2\times \right)$$$$\div\ protein\ concentration\ of\ the\ homogenate\ to\ be\ measured\ (\mathrm{g\ protein}/\mathrm{L})$$.

### The Gastrocnemius Wet Weight Ratio

Bilateral gastrocnemius muscles (*n* = 4) were stripped under anesthesia from rats in the SCI and GlyNAC+SCI groups 4 and 8 weeks post-injury. The weights of the freshly harvested gastrocnemius muscles were measured on electronic scales and the values were divided by the pre-harvest body weights of rats to obtain the gastrocnemius muscle wet weight ratio. The specific calculation method was gastrocnemius wet weight ratio = (left gastrocnemius weight + right gastrocnemius weight)/2/body weight of rats before sampling. The wet weight ratio of the gastrocnemius muscle was also calculated for the control group and the final values of the three groups were compared.

### Western Blot Analysis

Gastrocnemius muscle samples were homogenized in RIPA lysis buffer (ServiceBio, Wuhan, China) containing a 50× protease inhibitor cocktail (ServiceBio, Wuhan, China). The protein concentrations of the tissue samples were determined using a BCA protein quantification kit. After denaturing in a boiling water bath, the proteins were separated by SDS–polyacrylamide gel electrophoresis (SDS–PAGE), transferred to a PVDF membrane, blocked with 5% milk in TBST for 30 min at room temperature, and then incubated with primary antibody at 4 °C overnight with shaking. After primary antibody recovery and three washes with TBST, the membranes were incubated with secondary antibody (GB23303, HRP-conjugated goat anti-rabbit IgG, 1:5000; Servicebio) at room temperature for 30 min. The protein bands were revealed with a chemiluminescence reagent. The absolute OD and the relative OD were determined using AIWBwell analysis software.

### Immunofluorescence

Fresh rat gastrocnemius muscle tissues were cryopreserved at −80 °C and OCT-embedded 8 weeks after injury for sampling and sectioning. Frozen sections were rewarmed at room temperature; the tissue was circled with a histochemical pen, incubated with autofluorescence quenching agent (G1221, ServiceBio, Wuhan, China) for 5 min, and then rinsed. ROS stain (D7008, Sigma-Aldrich) was added dropwise within the circled area of the tissues, and the sections were incubated under light for 30 min (pH 7.4) three times, 5 min each. Then, the samples were counterstained with DAPI staining solution for 10 min at room temperature shielded from light, washed three times with PBS (pH 7.4), and sealed with an anti-fluorescence quenching agent. Finally, the ROS-positive cell rate, the area proportion, and the fluorescence intensity of ROS-positive cells were observed and analyzed with a fluorescence microscope (Eclipse C1, Nikon, Tokyo, Japan).

### Statistical Analysis

Statistical analysis was performed using SPSS version 19.0 (IBM, Armonk, NY, USA). Independent samples *t*-tests were used for comparisons between two groups and one-way ANOVA followed by Bonferroni’s *post hoc* test was employed for comparisons among multiple groups. Data are shown as means ± standard deviation. *P* values < 0.05 were considered significant. GraphPad Prism version 9.3.0.463 (LLC, San Diego, CA, USA) was used for graphing.

## Results

### GlyNAC Improved BBB Scores in SCI Model Rats

**Fig. 2:**
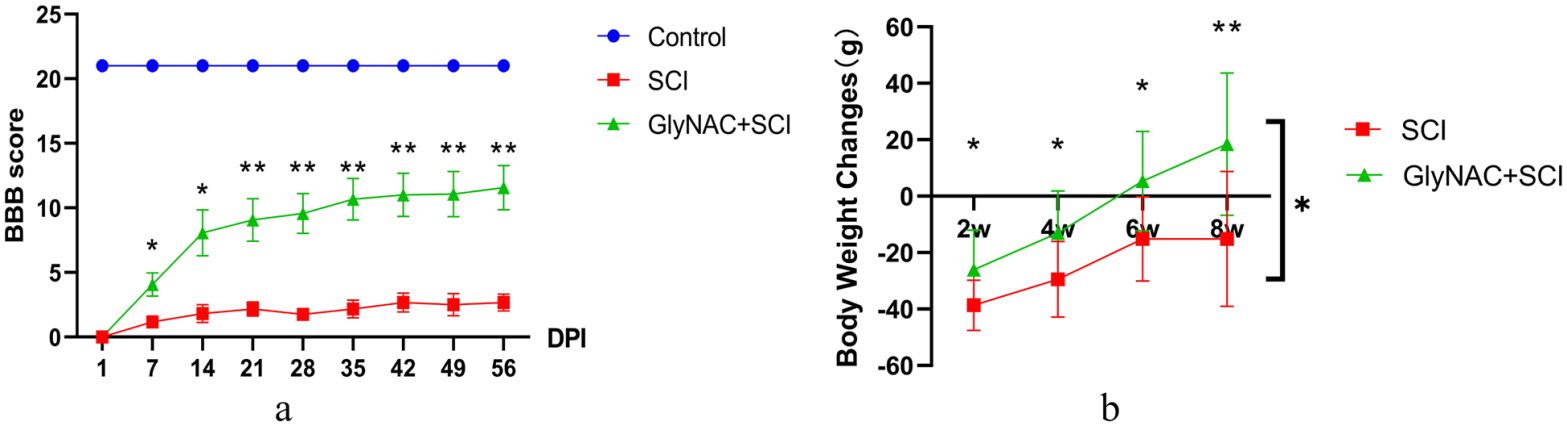
BBB scores and weight change. **a** BBB scores on day 1 and 1–8 weeks after modeling in the control, SCI, and GlyNAC+SCI groups. **b** Evaluation of weight changes in the SCI group and the GlyNAC+SCI group 2, 4, 6, and 8 weeks after modeling; weight change was calculated as the difference between the weights at the respective time points and the weight before modeling. *Indicates significant differences between the SCI and GlyNAC+SCI groups (one-way ANOVA, Bonferroni’s *post hoc* test). *P < 0.05, **P < 0.01. BBB, Basso–Beattie–Bresnahan; GlyNAC, glycine and N-acetylcysteine; SCI, spinal cord injury.

The changes in BBB scores of rats in the control, SCI, and GlyNAC+SCI groups over the 8 weeks after SCI are shown in Fig. 2a. SCI model rats developed complete paralysis of the lower limbs, with a BBB score of 0 on day 1, after which they slowly began to recover. The BBB scores of rats in the SCI and GlyNAC+SCI groups differed significantly by day 7 post-injury (*P* = 0.0161), and this difference gradually increased thereafter until week 8 (*P* = 0.0049). These results suggested that GlyNAC treatment improved the motor function of the model rats to some degree.

### GlyNAC Promoted Weight Recovery in SCI Model Rats

Figure 2b shows the body weight changes in rats of the SCI and GlyNAC+SCI groups. The differences between the body weights at 2, 4, 6, and 8 weeks and the pre-injury body weights were determined and the average of the differences in body weight change of the rats in each group at each time point was plotted as a line graph. A comparison was made between the two groups at each time point. The results showed that from the second week, the body weight of the GlyNAC+SCI group was significantly lower than that of the SCI group (*P* = 0.027); additionally, from week 6, the body weight of rats in the GlyNAC+SCI group tended to be greater than that observed pre-injury (*P* = 0.0116), whereas that of rats in the SCI group was still lower at week 8 than before modeling (*P* = 0.007). These findings indicated that GlyNAC can effectively promote weight recovery in rats after SCI, and further reflects that GlyNAC can improve the nutritional and metabolic status of SCI rats, as well as contribute to the maintenance of a good nutritional status.

### GlyNAC Improved Sensitivity to Thermal Pain in SCI Rats in the Short Term

**Fig. 3:**
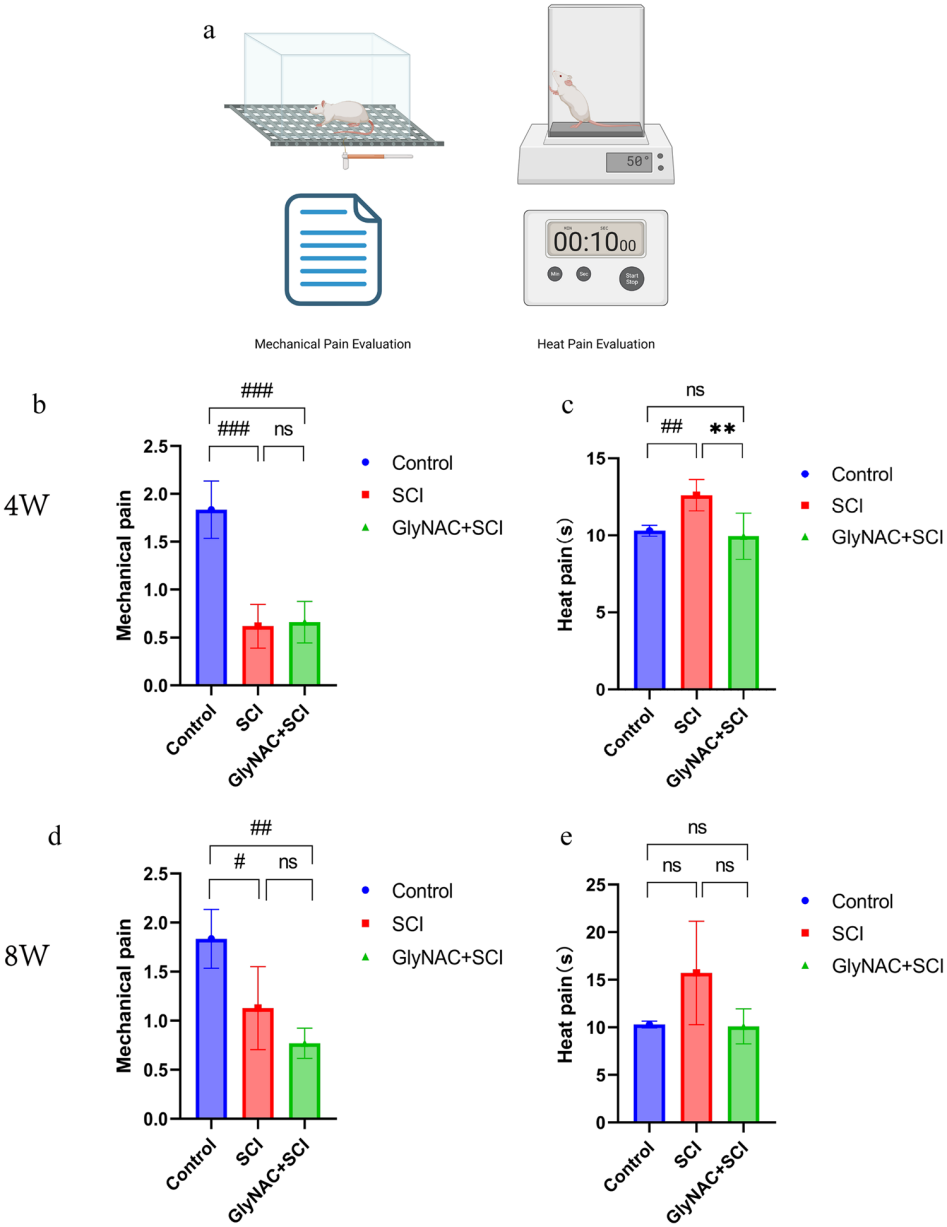
Thermal and mechanical pain assessment results. **a** Von Frey and thermal pain assessment methods. For the Von Frey assessment, rats from the control group, the SCI group, and the GlyNAC+SCI group were placed in a transparent cage and Von Frey hairs of different thicknesses were used to stimulate the soles of the hind paws of the rats through the metal mesh. Pain responses were recorded and the scores were calculated for the left and right hind paws, respectively. For thermal pain assessment, the hot plate method was used. The rats were placed on a plate heated to 50 °C and the time it took for the animals to lift or lick their feet was recorded. The average of three recordings was obtained for each (left and right) hind paw. **b**, **c** The results of the scores of the thermal and mechanical pain in the control group, the SCI group, and the GlyNAC+SCI group 4 weeks after modeling. **d**, **e** The results of the scores of the thermal and mechanical pain in the control group, SCI group, and GlyNAC+SCI group 8 weeks after modeling. * and ^#^ indicate significant differences between the SCI and GlyNAC+SCI groups, the control and SCI groups, and the control and GlyNAC+SCI groups; ns, not significant (one-way ANOVA, Bonferroni’s *post hoc* test). * or ^#^*P* < 0.05, ** or ^##^*P* < 0.01, *** or ^###^*P* < 0.001. GlyNAC, glycine and N-acetylcysteine; SCI, spinal cord injury.

The differences in thermal and mechanical pain responses between rats in the SCI and GlyNAC+SCI groups and those in the control group are shown in Fig. 3. The results showed that 4 weeks post-injury, the mechanical pain scores for rats in the SCI and GlyNAC+SCI groups were lower than those recorded for control animals (*P* = 0.0003; *P* = 0.0002); moreover, no difference in mechanical pain scores was recorded between the SCI and GlyNAC+SCI groups (*P* = 0.9682). However, rats in the GlyNAC+SCI group showed greater sensitivity to thermal pain stimulation compared with rats in the SCI group (*P* = 0.0016). After 8 weeks, the mechanical pain scores of rats in both the SCI and GlyNAC+SCI groups showed a significant decrease, with no significant difference being detected between the two groups (*P* = 0.2857); meanwhile, rats in the SCI group still showed a slower response to thermal hyperalgesia compared with rats in the GlyNAC+SCI group, but the difference was not significant (*P* = 0.0721). This suggested that GlyNAC may have a transient promotive effect on sensitivity to thermal pain in rats following SCI.

### GlyNAC Improved the Antioxidant Capacity of Spinal Cord Tissue in Model Rats

**Fig. 4:**
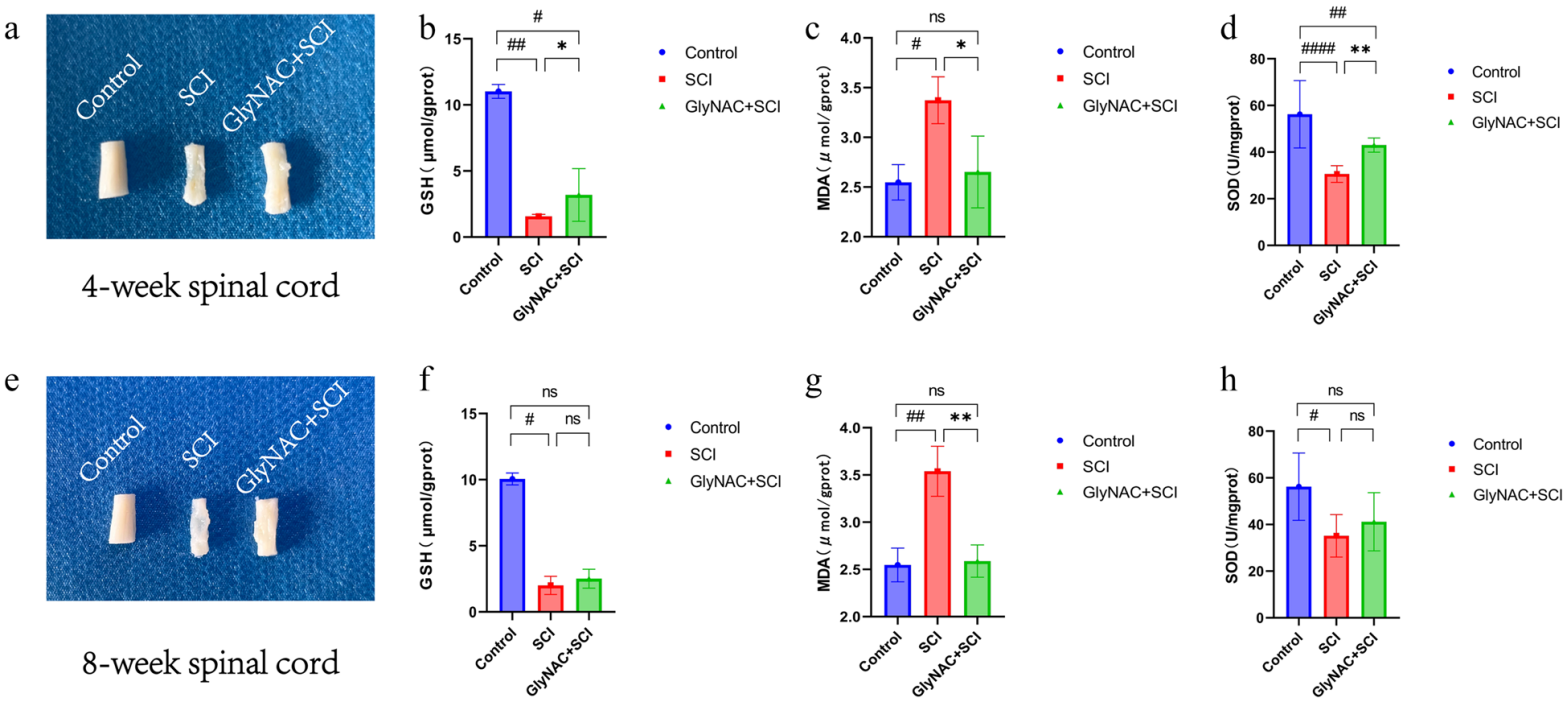
Morphological observation of the spinal cord and analysis of GSH, MDA, and SOD levels. **a**, **e** Morphological observation of the spinal cord in the injured area at 4 and 8 weeks after SCI. **b**–**d** Statistical analysis of GSH, MDA, and SOD levels in the control, SCI, and GlyNAC+SCI groups 4 weeks after modeling. **f**–**h** Statistical analysis of GSH, MDA, and SOD levels in the control, SCI, and GlyNAC+SCI groups 8 weeks after modeling. * and # indicate significant differences between the SCI and GlyNAC+SCI groups, the control and SCI groups, and the control and GlyNAC+SCI groups; ns denotes not significant (one-way ANOVA, Bonferroni’s *post hoc* test). * or ^#^*P* < 0.05, ** or ^##^*P* < 0.01, *** or ^###^*P* < 0.001, **** or ^####^*P* < 0.0001. GlyNAC, glycine and N-acetylcysteine; GSH, glutathione; MDA, malondialdehyde; SCI, spinal cord injury; SOD, superoxide dismutase.

The morphology of the spinal cord of rats in the control, SCI, and GlyNAC+SCI groups 4 and 8 weeks after SCI is shown in Fig. 4a and e. It can be seen that the spinal cord in the injured area of rats in the SCI and GlyNAC+SCI groups showed different degrees of atrophy and glial scarring at both time points. However, in the GlyNAC+SCI group, the spinal cord in the middle region of the injury was morphologically more intact, and atrophy and glial scarring were less severe; this implied that GlyNAC exerts a neuroprotective effect after SCI, at least to some extent. The levels of GSH, MDA, and SOD in rats of the three groups were measured at 4 and 8 weeks post-injury. At the 4-week sampling point, the GSH levels in both the SCI and GlyNAC+SCI groups were markedly decreased compared with that in the control group (*P* = 0.0008; *P* = 0.0242); however, the level of GSH in the GlyNAC+SCI group was still significantly higher than that in the SCI group (*P* = 0.0242). Additionally, the level of MDA in the SCI group was significantly higher than that in both the control group (*P* = 0.0048) and the GlyNAC+SCI group (*P* = 0.0107). Meanwhile, SOD activity was significantly decreased in both the SCI (*P* = 0) and the GlyNAC+SCI (*P* = 0.0045) groups relative to that recorded in the control group; nevertheless, SOD activity was higher in the GlyNAC+SCI group than in the SCI group (*P* = 0.0045) (Fig. 4b–d). At 8 weeks post-injury, the GSH levels in SCI model rats were significantly decreased in comparison with those in control animals (*P* = 0.0152); however, no significant difference in GSH content was detected between rats in the GlyNAC+SCI group and those in the SCI group (*P* = 0.4566). The MDA level in the SCI group was significantly higher than that in both the control group (*P* = 0.0002) and the GlyNAC+SCI group (*P* = 0.0003). Finally, SOD activity was significantly decreased in the SCI group (*P* = 0.046) compared with that in the control group; in contrast, there was no significant difference in SOD activity between the GlyNAC+SCI group and the SCI group (*P* = 0.6737) (Fig. 4f–h). The above results indicated that GlyNAC can, to a certain extent, improve antioxidant capacity, reduce oxidative stress-induced damage, and exert neuroprotective effects in spinal cord tissues of rats after SCI.

### GlyNAC Improved the Antioxidant Capacity of Skeletal Muscle Tissue in SCI Rats

**Fig. 5:**
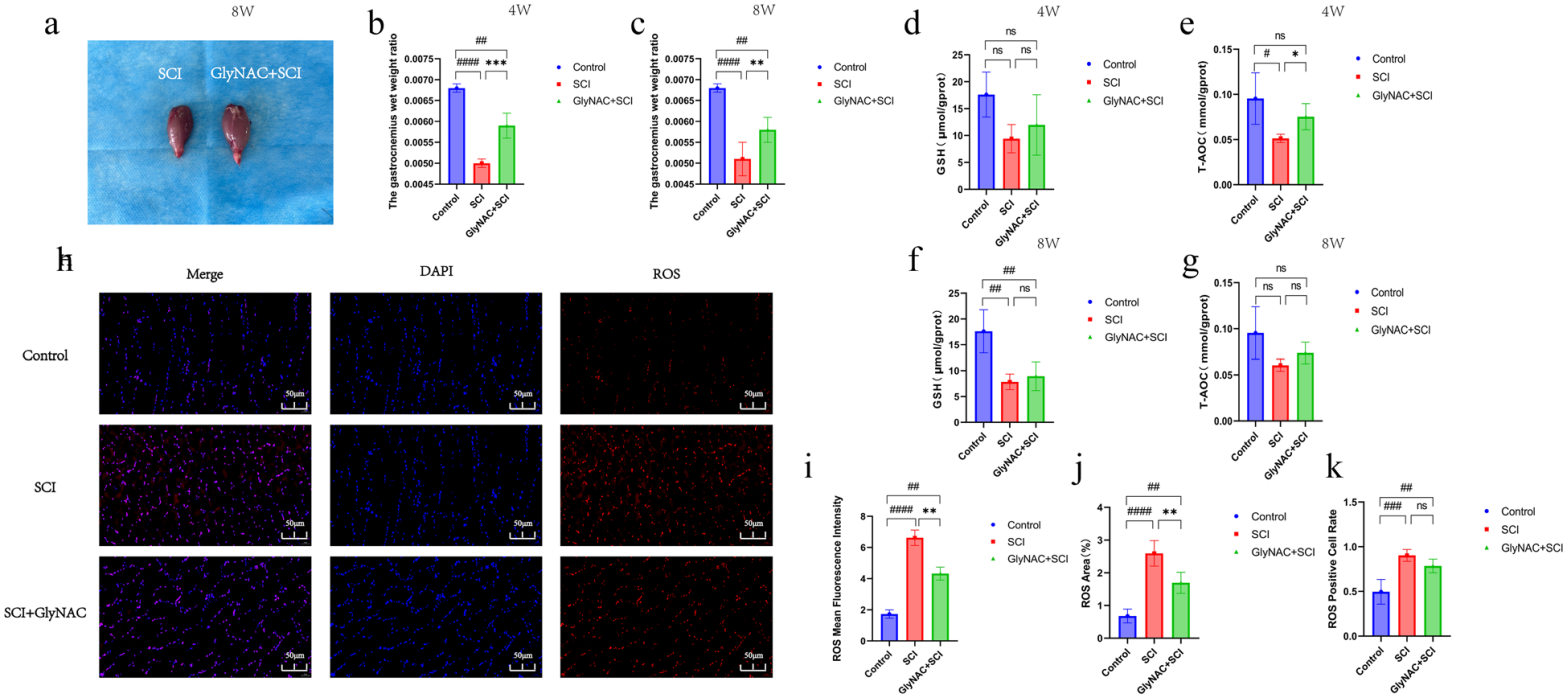
Morphological observation of the gastrocnemius muscle, gastrocnemius muscle wet weight/body weight ratio, and assessment of GSH and ROS levels and T-AOC in gastrocnemius muscle. **a**, **e** Morphological observation of the gastrocnemius muscle in the SCI and GlyNAC+SCI groups 8 weeks after SCI. **b**, **c** Results of the statistical analysis of the gastrocnemius muscle wet weight ratio in the three groups 4 and 8 weeks after modeling. **d**–**g** Statistical analysis of GSH levels and T-AOC in the three groups 4 and 8 weeks after modeling. **h** Immunofluorescence staining of ROS in gastrocnemius muscle of the three groups 8 weeks after modeling. **i**–**k** Statistical analysis of the intensity of ROS immunofluorescence, the percentage of ROS area, and the ROS-positive cell rate in gastrocnemius muscle of the three groups 8 weeks after modeling. * and # indicate significant differences between the SCI and GlyNAC+SCI groups, the control and SCI groups, and the control and GlyNAC+SCI groups; ns indicates not significant (one-way ANOVA, Bonferroni’s *post hoc* test). * or ^#^*P* < 0.05, ** or ^##^*P* < 0.01, *** or ^###^*P* < 0.001, **** or ^####^*P* < 0.0001. GlyNAC, glycine and N-acetylcysteine; GSH, glutathione; T-AOC, total antioxidant capacity; ROS, reactive oxygen species; SCI, spinal cord injury.

Figure 5a shows the differences in the morphology of the gastrocnemius muscle between the SCI group and the GlyNAC+SCI group 8 weeks post-injury. A morphological observation indicated that the gastrocnemius muscle of rats in the GlyNAC+SCI group was fuller and appeared to be larger. Accordingly, we next determined the gastrocnemius wet weight ratio in the control, SCI, and GlyNAC+SCI groups at 4 and 8 weeks after SCI. We found that compared with the control group, the gastrocnemius wet weight ratios of the SCI and GlyNAC+SCI groups were significantly decreased at both 4 (*P* = 0.0000032919; *P* = 0.0011) weeks and 8 (*P* = 0; *P* = 0.0045) weeks after injury. Additionally, the gastrocnemius wet weight ratio in the SCI group was significantly lower than that in the GlyNAC+SCI group at 4 weeks (*P* = 0.0006) and 8 weeks (*P* = 0.0045) post-injury (Fig. 5b, c). This suggested that GlyNAC has a protective effect against skeletal muscle atrophy. Next, we further examined the oxidative stress-related indexes in the gastrocnemius muscle in the three groups. At 4 weeks post-SCI, no significant difference in GSH concentration was detected among the three groups (control vs SCI, *P* = 0592; control vs SCI+GlyNAC, *P* = 0.2091; SCI vs SCI+GlyNAC, *P* = 0.6916). However, T-AOC in the SCI group was significantly decreased compared with that in the control group (*P* = 0.0102) and the GlyNAC+SCI group (*P* = 0.0498) (Fig. 5d, e); at the 8-week sampling time point, meanwhile, the GSH concentration was significantly lower in the SCI group (*P* = 0.0035) and the GlyNAC+SCI group (*P* = 0.0074) than in the control group; there was no significant difference in T-AOC among the three groups (Fig. 5f, g). Meanwhile, we undertook an immunofluorescence staining analysis of ROS levels in gastrocnemius muscle tissue at 8 weeks post-SCI (Fig. 5h). ROS fluorescence intensity, the percentage of ROS-positive area, and the ROS-positive cell rate were compared among the three groups. The results showed that ROS fluorescence intensity was significantly higher in the SCI (*P* = 0) and GlyNAC+SCI (*P* = 0.0036) groups than in the control group and was also significantly higher in the SCI group than in the GlyNAC+SCI group (*P* = 0.0076) (Fig. 5i). The percentage of ROS-positive area was significantly higher in the SCI group (*P* = 0) and the GlyNAC+SCI group (*P* = 0.0036) than in the control group and was significantly higher in the SCI group than in the GlyNAC+SCI group (*P* = 0.0076) (Fig. 5j). The ROS-positive cell rate was significantly higher in the SCI (*P* = 0.0007) and GlyNAC+SCI (*P* = 0.0064) groups than in the control group; however, no significant difference in the ROS-positive cell rate was recorded between the SCI and the GlyNAC+SCI groups (*P* = 0.2658) (Fig. 5k). The above results suggested that GlyNAC administration exerts a protective effect against oxidative stress-induced damage in gastrocnemius muscle tissue after SCI.

### Investigation of the Effect of GlyNAC on the Expression of Peroxisome Proliferator-Activated Receptor-Gamma Coactivator-1Alpha (PGC-1α) in Skeletal Muscle Tissues of SCI Rats

**Fig. 6:**
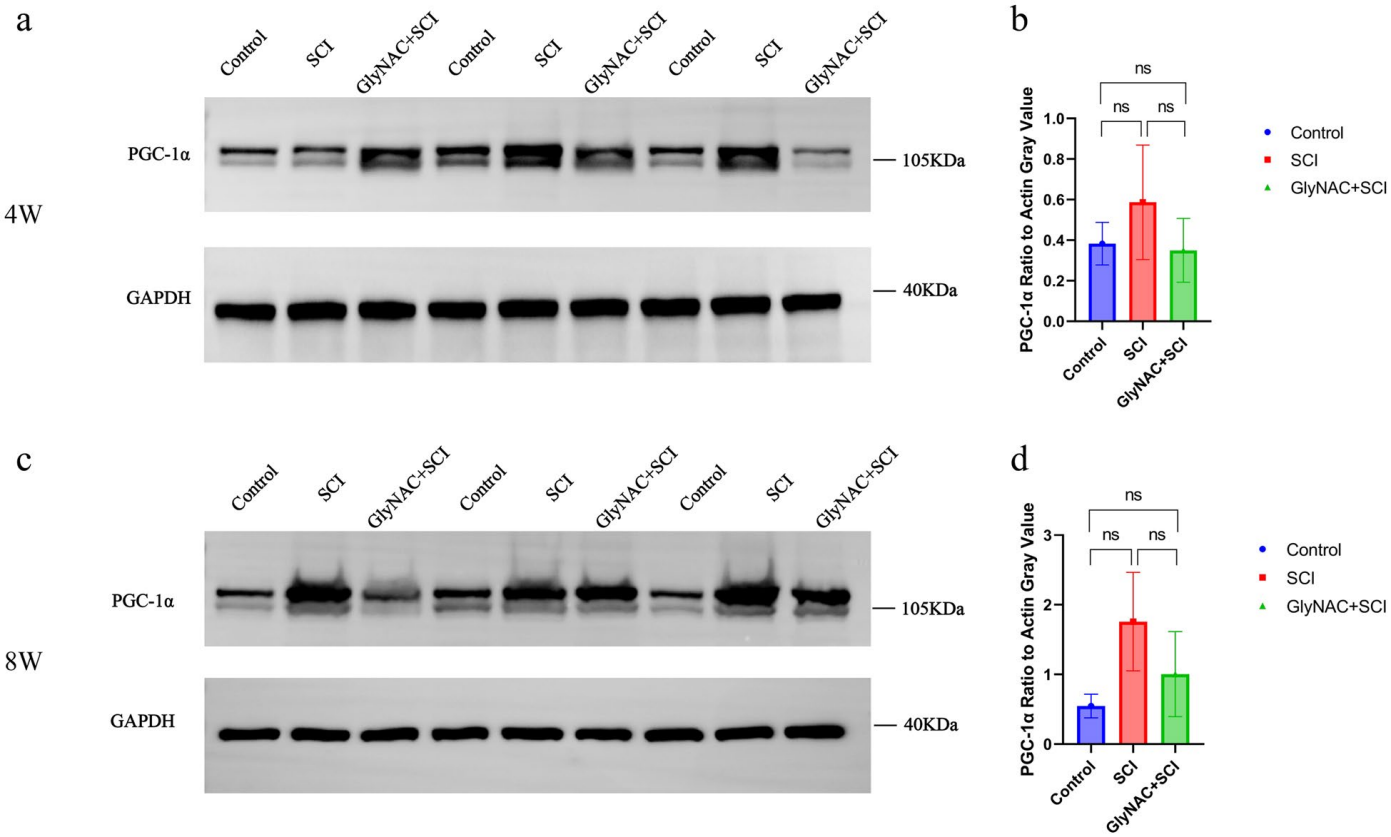
Results of the statistical analysis of PGC-1α western blotting bands and protein expression in gastrocnemius muscle. **a**, **c** Bands of PGC-1α protein expression in gastrocnemius muscle in the three groups 4 and 8 weeks after modeling. **b**, **d** Results of the statistical analysis of PGC-1α protein expression levels in gastrocnemius muscle in the three groups 4 and 8 weeks after modeling; ns denotes not significant (one-way ANOVA, Bonferroni’s *post hoc* test). PGC-1α, peroxisome proliferator-activated receptor-gamma coactivator-1alpha.

PGC-1α expression in gastrocnemius muscle of the control, SCI, and GlyNAC+SCI groups at 4 and 8 weeks post-SCI as determined by western blot is shown in Fig. 6a and c. Statistical analysis of protein levels revealed that there was no significant difference in the expression level of PGC-1α in skeletal muscle tissue between the three groups at either 4 (control vs SCI, *P* = 0.4595; control vs SCI+GlyNAC, *P* = 0.9764; SCI vs SCI+GlyNAC, *P* = 0.364) or 8 (control vs SCI, *P* = 0.128; control vs SCI+GlyNAC, *P* = 0.6638; SCI vs SCI+GlyNAC, *P* = 0.3856) weeks post-injury (Fig. 6b, d).

## Discussion

**Fig. 7:**
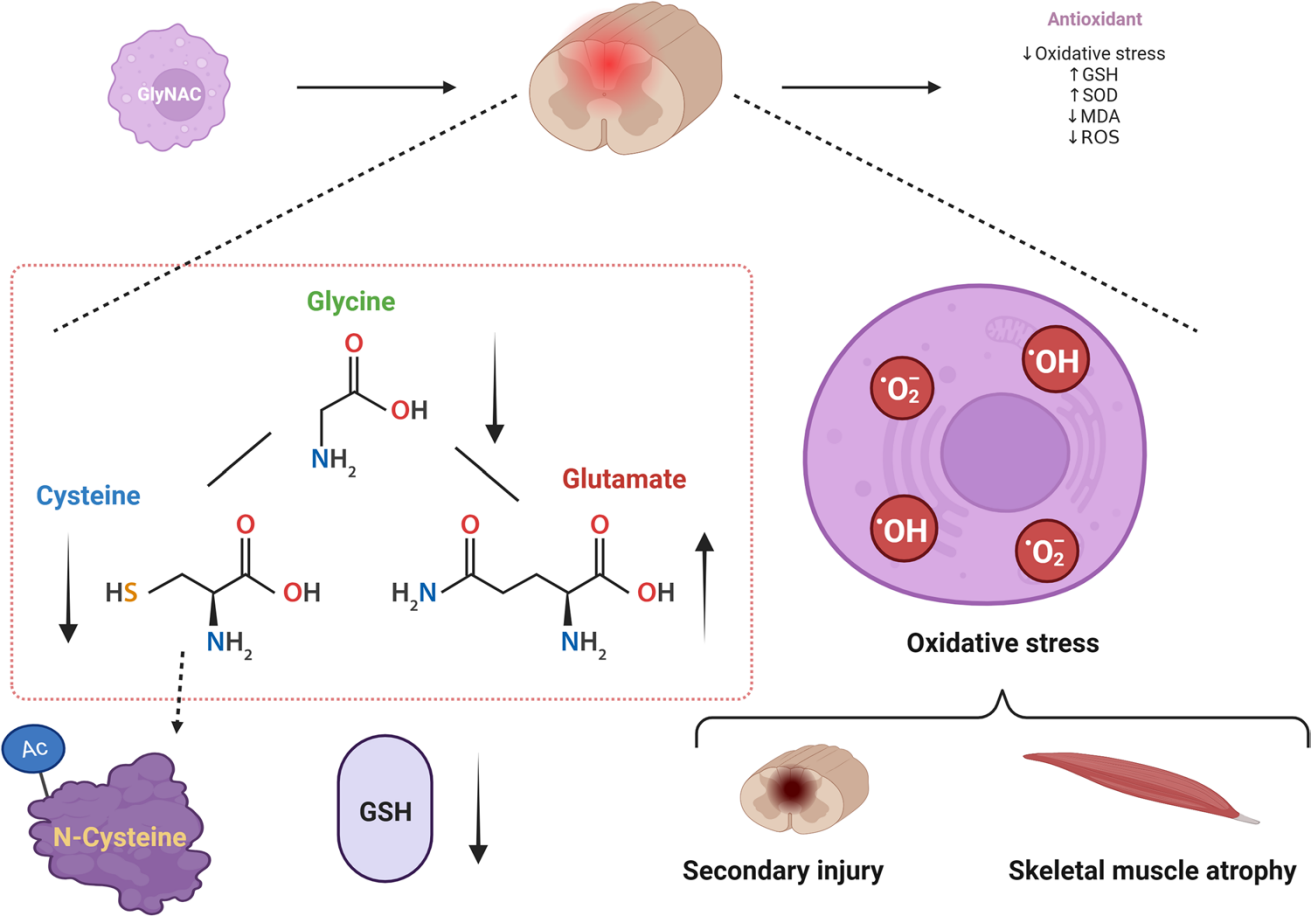
After SCI, the level of GSH decreases, and at the same time, the raw material for GSH synthesis in the spinal cord will show an increase in the level of glutamate, and a decrease in the level of glycine and cysteine, which leads to oxidative stress-induced damage, resulting in secondary spinal cord injury and skeletal muscle atrophy. GlyNAC can be used as an antioxidant to fight against oxidative stress injury, which can contribute to the increase in the level of antioxidant molecules, such as GSH, SOD, and other antioxidant molecules, and the decrease in the level of MDA and ROS. GlyNAC can be used as an antioxidant to resist oxidative stress injury, increase the level of antioxidant molecules such as GSH and SOD, and decrease the level of MDA and ROS. SCI, spinal cord injury; GSH, glutathione; GlyNAC, glycine and N-acetylcysteine; SOD, superoxide dismutase; MDA, malondialdehyde; ROS, reactive oxygen species.

Glycine is an important inhibitory neurotransmitter [14] and has also been found to exert cytoprotective effects under conditions of glucose–oxygen deprivation [17]. Ascher *et al*. [18] found that glycine could help preserve skeletal muscle function and prevent ischemia–reperfusion injury in skeletal muscle cells under hypoxic and ischemic conditions. Glycine was also demonstrated to inhibit the deleterious effects of oxidative injury on skeletal muscle and help counteract the inflammatory response that leads to skeletal muscle atrophy [19]. Glycine levels in spinal cord tissue were reported to increase 1 day after SCI and then decrease by 8 weeks after injury [20]. Meanwhile, glycine can reportedly also restore the anabolic response to leucine under conditions of skeletal muscle wasting [21]. Margaritelis *et al*. identified a positive correlation between dietary intake of cysteine and GSH concentrations [11]. NAC is a better source of the antioxidant sulfhydryl than cysteine and has demonstrated stronger antioxidant activity [22]. NAC was also reported to possess neuroprotective properties [23] and inhibit the expression of skeletal muscle atrophy-related proteins by neutralizing the toxic effects of ROS [24] (Fig. 7).

Glycine is also documented as being rate-limiting in GSH synthesis [25, 26]. When NAC was given alone, it accelerated senescence and shortened the lifespan of the nematode worm *Caenorhabditis elegans* [27]. Recent studies have found that the GlyNAC complex can correct GSH deficiency, counteract oxidative stress, correct type II diabetes, reduce insulin resistance, enhance muscle strength, and improve cognitive function in older adults, suggesting that it has anti-aging effects [28–30]. However, no study to date has assessed the effects of GlyNAC in SCI. Here, we explored whether the exogenous supplementation of GlyNAC, a precursor complex of GSH, could promote the synthesis of GSH *in vivo*, and sought to identify the putative intrinsic molecular mechanism. We hypothesized that by combining the advantages of glycine and NAC, GlyNAC would counteract oxidative stress-induced damage after SCI and play a robust protective role in the injured spinal cord and skeletal muscle (Fig. 7).

A cascade involving edema, an inflammatory response, ROS/reactive nitrogen species (RNS) production, glutamate-mediated toxic effects, neuronal apoptosis, ferroptosis, and glial scarring occurs after SCI [31–33]. Oxidative injury plays a major role in the pathophysiology of SCI [5] by inducing lipid peroxidation in the spinal cord and skeletal muscle tissues. We found that SCI rats administered exogenous GlyNAC showed a significant increase in BBB scores, which, to some extent, reflected a recovery of motor function. Four weeks after surgery, the thermal pain sensitivity of the rats was partially restored with GlyNAC treatment, suggesting that GlyNAC has a transient ameliorative effect on temperature sensitization in rats post-SCI. Additionally, the spinal cord of rats administered GlyNAC had a more complete morphology and less severe atrophy compared with that of the naturally recovered spinal cord, suggesting that GlyNAC has a neuroprotective effect on the spinal cord of rats after injury.

Meanwhile, we also explored the antioxidant effects of GlyNAC in the spinal cord at the molecular level. We found that after 4 weeks of GlyNAC supplementation, the GSH level was increased in damaged spinal cord tissues and was significantly higher than that seen in spinal cord tissues that had recovered naturally from the same injury. However, once GlyNAC intake stopped after 4 weeks, the GSH level in the GlyNAC+SCI and SCI groups displayed a similar trend, even though it remained higher in the former, suggesting that the maintenance of the GSH level may have been dependent on GlyNAC intake.

The membranes of spinal cord cells are also damaged by free radicals after SCI. This involves the removal of electrons from lipids, resulting in lipid peroxidation and, consequently, cellular damage [34]. Our results showed that at both 4 and 8 weeks post-injury, the levels of MDA, widely used as an indicator of lipid peroxidation, were significantly higher in the SCI group than in the GlyNAC+SCI group or the control group; in contrast, there was no significant difference in MDA levels between the GlyNAC+SCI and control groups. This indicated that GlyNAC can protect against lipid peroxidation in the injured spinal cord, which is consistent with our morphological observations.

SOD is an important component of the body’s antioxidant defense system, protecting cells from free radicals and, consequently, oxidative stress [35]. However, because its half-life in circulation is only approximately 6 min, SOD cannot be directly administered as a therapeutic strategy [36]. The results of our study demonstrated that SOD activity in spinal cord tissues decreased significantly after SCI, but was significantly higher in the GlyNAC+SCI group than in the SCI group 4 weeks post-injury; after 8 weeks, meanwhile, no significant difference in SOD activity was observed between the two groups. This suggests that SOD activity is also negatively affected in damaged spinal cord tissues during the chronic phase of SCI, which leads to a decrease in the antioxidant capacity of the spinal cord, and is an important factor in the occurrence of oxidative stress-induced injury. However, our findings indicated that GlyNAC may have enhanced SOD activity in damaged spinal cord tissue, thus protecting against oxidative injury.

After SCI, skeletal muscle undergoes severe atrophy due to the loss of innervation. Changes such as apoptosis and protein degradation in skeletal muscle cells can lead to multiple secondary metabolic dysfunctions, including glucose intolerance, type II diabetes mellitus, and insulin resistance [37, 38]. Skeletal muscle also suffers oxidative injury after SCI [39]. In this study, we selected the gastrocnemius muscle, a critical muscle innervated by spinal segments below the site of injury in the lower extremity, as the study subject. We found that the gastrocnemius wet weight ratio in the SCI group was significantly decreased at 4 and 8 weeks compared with that in both the control and GlyNAC+SCI groups. Additionally, we found that gastrocnemius muscle atrophy was more pronounced in the SCI group than in the GlyNAC+SCI group post-SCI. Combined, these results support that GlyNAC can, to some extent, protect against skeletal muscle atrophy.

Next, we examined the levels of oxidative stress-related molecules in the gastrocnemius muscle. The results demonstrate that, during the chronic phase of SCI, the GSH level in skeletal muscle decreases, rendering the skeletal muscle vulnerable to oxidative stress-induced damage and, consequently, atrophy. Meanwhile, whether GlyNAC supplementation can enhance GSH levels in skeletal muscle after SCI remains to be further explored. We also determined the T-AOC as a representative indicator of the antioxidant capacity of skeletal muscle and found that at 4 weeks, T-AOC was significantly decreased in SCI rats relative to that in the control and GlyNAC+SCI groups. SCI leads to ROS accumulation in skeletal muscle [40], resulting in oxidative injury, a key contributing factor to skeletal muscle atrophy [41]. The results of the immunofluorescence staining in the gastrocnemius muscle at 8 weeks showed that ROS accumulation and release increased after SCI, the ROS-positive cell rate increased significantly, the percentage of ROS-positive area also increased, and the immunofluorescence intensity was significantly enhanced. Meanwhile, immunofluorescence intensity and the percentage of ROS-positive area were lower in the GlyNAC+SCI group than in the SCI group, which suggests that GlyNAC intake can delay skeletal muscle atrophy by enhancing the antioxidant capacity of skeletal muscle, including ROS-scavenging ability.

PGC-1α is expressed at high levels in skeletal muscle and has an important role in skeletal muscle repair by inducing mitochondrial genesis and angiogenesis and promoting energy metabolism [42–44]. After SCI, the expression level of PGC-1α in skeletal muscle decreases significantly [45], and indirectly contributes to the expression levels of human dystrophin (Atrogin-1) and muscle-specific RING finger protein 1 (MuRF1), resulting in skeletal muscle atrophy [46]. However, in our study, no significant changes in PGC-1α levels were seen in the gastrocnemius muscle at both 4 and 8 weeks after SCI. This is more in line with the findings of Scholpa *et al*. [47]: Their study found that when intervening in post-SCI skeletal muscle atrophy by means of a PGC-1α activator, a significant difference in the level of PGC-1α expression appeared in skeletal muscle undergoing the intervention in the early stages of the injury, with no significant difference at the chronic stage. In addition to contributing to the relatively small sample size, we hypothesize that GlyNAC has an effect on PGC-1α levels mainly in the early stage of injury to promote functional recovery, but PGC-1α levels in skeletal muscle remain relatively stable in the chronic phase.

GSH cannot be supplemented through oral intake because it is highly digestible in the gut, which reduces its effectiveness [48]. Therefore, in the present study, we employed GlyNAC as the intervention to investigate the therapeutic effects of glycine, NAC, and GSH in SCI from the perspective of oxidative injury, behavioral manifestations, and levels of molecular markers. Unlike previous studies on changes in oxidative stress-related molecules in acute and subacute phases of SCI, we focused on changes in the concentrations of oxidative stress-related molecules in the spinal cord and skeletal muscle in the chronic phase. However, the long-term efficacy of GlyNAC following SCI was not determined in this study because of time limitations, and it is thus not possible to identify a more appropriate intervention period. Meanwhile, in this study, we compared the gastrocnemius wet weight ratio among the three groups, and gastrocnemius muscle was used as the selected target to infer the consistent effect of GlyNAC on the involved skeletal muscles. For a comprehensive analysis, other skeletal muscles should also be used as the research target. In this study, the effectiveness of GlyNAC was demonstrated only in rats. Further studies are needed to confirm its safety and efficacy and determine a safe and effective dose for clinical use in SCI.

## Conclusion

In summary, the antioxidant capacity and GSH levels in both the spinal cord and skeletal muscle showed a significant decrease in the chronic period after SCI, which resulted in lipid peroxidation-mediated injury in the spinal cord. However, GlyNAC exerted a protective effect against the resulting oxidative injury in the spinal cord and skeletal muscle tissue after SCI, delayed skeletal muscle atrophy, and promoted the functional recovery of the rats. This suggests that GlyNAC can be added to the diets of patients with SCI and has value and potential for use as a nutritional therapy.

## Data Availability

The datasets and materials used and/or analyzed during the current study are available from the corresponding author on reasonable request.

## Abbreviations

SCI: Spinal cord injury
GlyNAC: Glycine and N-acetylcysteine
OS: Oxidative stress
ROS: Reactive oxygen species
GSH: Glutathione
SD: Sprague–Dawley
BBB: Basso–Beattie–Bresnahan
SOD: Superoxide dismutase
MDA: Malondialdehyde
T-AOC: Total antioxidant capacity
PGC-1α: Peroxisome proliferator-activated receptor-gamma coactivator-1alpha
RNS: Reactive nitrogen species
Atrogin-1: Human dystrophin
MuRF1: Muscle-specific RING finger protein 1
